# Development and validation of a risk nomogram model for predicting peripheral neuropathy in patients with type 2 diabetes mellitus

**DOI:** 10.3389/fendo.2024.1338167

**Published:** 2024-04-29

**Authors:** Lingguang Luo, Xinping Long, Cheng Cheng, Qian Xu, Jing Li

**Affiliations:** ^1^ Department of Endocrinology and Metabolism, The People’s Hospital of Laibin, Guangxi, China; ^2^ Department of Nephrology, The People’s Hospital of Laibin, Guangxi, China; ^3^ Department of Endocrinology and Metabolism, Suqian First Hospital, Jiangsu, China

**Keywords:** diabetic peripheral neuropathy, risk factor, model, prediction, nomogram

## Abstract

**Objective:**

Diabetic peripheral neuropathy frequently occurs and presents severely in individuals suffering from type 2 diabetes mellitus, representing a significant complication. The objective of this research was to develop a risk nomogram for DPN, ensuring its internal validity and evaluating its capacity to predict the condition.

**Methods:**

In this retrospective analysis, Suqian First Hospital’s cohort from January 2021 to June 2022 encompassed 397 individuals diagnosed with T2DM. A random number table method was utilized to allocate these patients into two groups for training and validation, following a 7:3 ratio. By applying univariate and multivariable logistic regression, predictive factors were refined to construct the nomogram. The model’s prediction accuracy was assessed through metrics like the ROC area, HL test, and an analysis of the calibration curve. DCA further appraised the clinical applicability of the model. Emphasis was also placed on internal validation to confirm the model’s dependability and consistency.

**Results:**

Out of 36 evaluated clinicopathological characteristics, a set of four, duration, TBIL, TG, and DPVD, were identified as key variables for constructing the predictive nomogram. The model exhibited robust discriminatory power, evidenced by an AUC of 0.771 (95% CI: 0.714-0.828) in the training cohort and an AUC of 0.754 (95% CI: 0.663-0.845) in the validation group. The congruence of the model’s predictions with actual findings was corroborated by the calibration curve. Furthermore, DCA affirmed the clinical value of the model in predicting DPN.

**Conclusion:**

This research introduces an innovative risk nomogram designed for the prediction of diabetic peripheral neuropathy in individuals suffering from type 2 diabetes mellitus. It offers a valuable resource for healthcare professionals to pinpoint those at elevated risk of developing this complication. As a functional instrument, it stands as a viable option for the prognostication of DPN in clinical settings.

## Introduction

T2DM, a widespread chronic metabolic condition, is predominantly identified by elevated levels of glucose in the blood. This condition stems from a dual complication: the body’s resistance to insulin and a deficiency in insulin production (1). According to the International Diabetes Federation’s reports up to September 2021, the global incidence of diabetes in 2019 was estimated at 9.3% (involving 463 million people), with forecasts suggesting a rise to 10.2% (578 million people) by 2030 and further to 10.9% (encompassing 700 million people) by 2045. Notably, approximately 90% of these cases are identified as T2DM (2). Such staggering figures indicate a substantial impact on both healthcare infrastructures and the lives of those diagnosed with the disorder.

Patients with T2DM frequently experience DPN, a serious complication marked by a gradual reduction in nerve function from the extremities inward (3). This condition is prevalent in 30-50% of T2DM patients, leading to significant consequences like physical impairments and potentially intense neuropathic pain (3–5). Beyond negatively affecting life quality and heightening the likelihood of minor injuries, which could escalate to severe infections or even amputations (6), The presence of DPN is significantly linked to elevated mortality rates due to various causes, including cardiovascular issues, among individuals with diabetes (7). Despite this, awareness of DPN among diabetic individuals remains inadequate. There is a clear and urgent necessity for an inclusive, easily navigable tool that consolidates identified risk factors for DPN in T2DM, facilitating precise risk assessment for each patient.

Nomograms are increasingly recognized as effective tools in clinical risk assessment, given their proficiency in integrating various variables into a cohesive and visually comprehensible instrument. Our hypothesis is that a model based on nomograms, encompassing diverse clinical, demographic, and lab parameters, will develop into a comprehensive predictive model, effectively estimating DPN risk. The objective of this study is to bridge a significant research gap by creating and validating a nomogram for DPN prediction in T2DM patients, facilitating the early identification of patients at high risk and offering a dependable guide for initial clinical interventions.

## Materials and methods

### Study design and population

**Figure 1:**
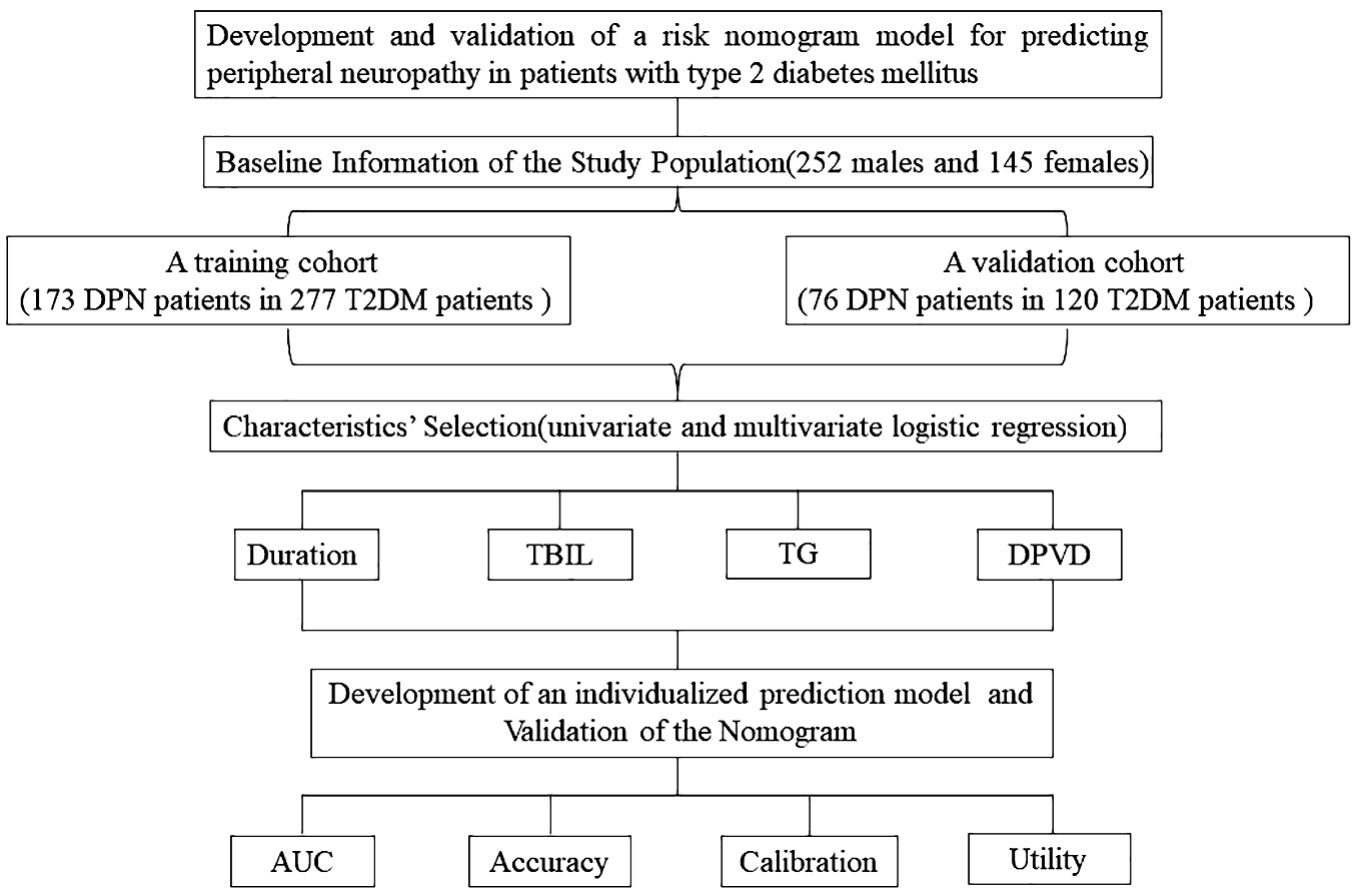
Flow chart of our study.

This study aimed to develop and corroborate a nomogram for evaluating DPN risk in T2DM patients. Conducted as a cross-sectional analysis, it encompassed 397 Chinese individuals diagnosed with T2DM, who were enrolled at Suqian First Hospital from January 2021 through June 2022 (**Figure 1**). Inclusion criteria stipulated that participants must be a minimum of 18 years of age and diagnosed with T2DM, adhering to the criteria set forth by the American Diabetes Association in 2021 (8), and capable of independent communication. Exclusion criteria encompassed patients with incomplete clinical records or other neuropathic conditions not related to diabetes, such as neuropathies due to systemic toxicity (alcohol abuse), neurotoxic medications (chemotherapy), vitamin B12 deficiency, hypothyroidism, renal diseases, specific cancers (multiple myeloma, bronchogenic carcinoma), infections like HIV, chronic inflammatory demyelinating neuropathy, genetic neuropathies, and vasculitis-related neuropathies (9).

### Neuropathy assessment

Diagnosis criteria included (1): confirmed diabetes history or signs of abnormal glucose metabolism; (2) onset of neuropathy concurrent with or following diabetes diagnosis; (3) definitive clinical or electrophysiological evidence of diabetic-related peripheral nerve damage; (4) exclusion of other peripheral neuropathy causes. Comprehensive neurological examinations were performed on all T2DM patients by expert neurologists for detailed evaluation. Experienced technicians conducted electrophysiological tests using electromyography equipment. Indicators of DPN included abnormal nerve conduction velocity or scores exceeding 7 on the MNSIQ (10). Assessment included recording the amplitude, distal latency, and velocity of nerve conduction for the compound muscle action potentials in the ulnar, median, tibial, and common peroneal nerves. Moreover, evaluations of the amplitude and nerve conduction velocity were conducted for the sensory nerve action potentials in the ulnar, median, and superficial peroneal nerves. Reference values were based on data from the healthy Chinese population. Doctors determined abnormal nerve conduction by identifying irregularities in one or more characteristics across at least two nerves.

### Diabetic peripheral vascular disease

Diagnosis can be made if one or more of the following clinical symptoms occur and at least two of the three tests are positive based on comprehensive indicators:

1) Symptoms: (1) Intermittent claudication; (2) Resting pain; (3) Limb pain (with or without sensory abnormalities, paralysis, no pulse, pale skin); (4) Skin ulcer.

2) Inspection: (1) Percutaneous oxygen partial pressure measurement<40mmHg; (2) In ankle brachial index measurement, ABI<0.9 or ABI>1.3; (3) Ultrasound scan indicates vascular lesions.

### Data collection

The study encompassed a comprehensive collection of initial clinical features, including BMI, WBC, MONO, LYM, NEUT, RBC, Hb, PLT, FT3, FT4, TSH, PT, APTT, PTINR, HbA1c, TC, TBIL, DBIL, AST, ALT, Scr, BUN, ALB, FPG, ALP, HDL-C, LDL-C, CHE, UA, TG, along with conditions like DPVD, DKD, and DPN.

### Training and validation of the nomogram

In this study, 397 individuals diagnosed with T2DM were randomly assigned into two groups. The first group, comprising 277 participants, served as the training set, while the second group, consisting of 120 individuals, functioned as the validation set. This distribution followed a 7:3 ratio (**Figure 1**). The study employed both univariate and multivariate logistic regression methods to pinpoint key variables. The performance of these methods was appraised through the analysis of ROC curves. The predictive precision of the models for DPN was determined using the AUC. Calibration plots and the Hosmer-Lemeshow test were employed to confirm the nomogram’s accuracy. Additionally, we employed R software (version 4.1.3) for DCA to assess the nomogram models’ net benefit at varying probability thresholds in the datasets.

### Statistical methods

The statistical analysis in this research utilized R software (version 4.2.1) alongside RStudio (version 2021.09.02). For analyzing continuous variables, their mean and standard deviation were calculated and represented as mean ± SD. In contrast, categorical variables were depicted using their frequency and percentage, denoted as n (%). Comparison between groups was performed using the Student’s t-test for continuous data and the χ² test for categorical data. When variables showed non-normal distribution, median and interquartile ranges were applied, with the Wilcoxon rank-sum test for comparing groups. We conducted both univariate and multivariate logistic regression analyses. Significant risk factors (P ≤ 0.05) were identified using forward-backward stepwise regression for developing a nomogram model. The ‘rms’ package facilitated the construction of the nomogram and calibration curve. Evaluation of characteristics was done using OR with 95% CI. P-values were computed bidirectionally for statistical relevance. The predictive model’s precision was assessed by ROC curve analysis, calibration plot generation, and DCA using the training and validation cohort data.

## Results

### Baseline information of the study population

This research included 397 subjects, comprising 252 males and 145 females. These participants were segregated into two cohorts: a training cohort consisting of 277 T2DM patients, of which 173 also had DPN, and a validation cohort comprising 120 individuals with T2DM, 76 of whom also presented with DPN. Proportionally, 62.45% of the training cohort and 63.33% of the validation cohort were diagnosed with both T2DM and DPN. Notably, the training and validation groups showed similar demographic and clinical profiles, with the exception of HbA1c levels, as detailed in **Table 1**.

**Table 1:** Baseline characteristics of patients in the training cohort and validation cohort.

Variables	Total (n = 397)	Training Cohort (n =277)	Validation Cohort (n = 120)	P value
sex,n,%				0.521
male	252(63.50)	173(62.50)	79(65.80)	
female	145(36.50)	104(37.50)	41(34.20)	
age(years)	55.21 ± 13.52	55.13 ± 13.53	55.21 ± 13.52	0.633
duration(years)	10.61 ± 7.84	9.88 ± 7.16	10.61 ± 7.84	0.401
BMI(kg/^2^)	25.60 ± 3.84	25.18 ± 4.09	25.60 ± 3.84	0.927
WBC(×10^9/L)	6.02 ± 1.50	6.16 ± 1.75	6.02 ± 1.50	0.094
MONO(×10^9/L)	0.46 ± 0.14	0.48 ± 0.16	0.47 ± 0.14	0.316
LYM(×10^9/L)	1.94 ± 0.60	1.91 ± 0.62	1.94 ± 0.60	0.841
NEUT(×10^9/L)	3.42 ± 1.10	3.60 ± 1.41	3.42 ± 1.10	0.056
RBC(×10^12/L)	4.58 ± 0.60	4.53 ± 0.60	4.59 ± 0.60	0.948
Hb(g/L)	138.26 ± 17.73	136.59 ± 18.61	138.26 ± 17.73	0.671
PLT(×10^9/L)	215.68 ± 63.42	208.29 ± 57.54	215.68 ± 63.42	0.361
25(OH)D3(ng/mL)	34.42 ± 23.36	33.45 ± 16.88	34.42 ± 23.36	0.204
FT3(pmol/L)	4.91 ± 1.50	4.58 ± 0.92	4.91 ± 1.50	0.804
FT4(pmol/L)	17.59 ± 4.06	16.89 ± 2.90	17.59 ± 4.06	0.107
TSH(mIU/L)	2.04(1.33-2.89)	1.95(1.35-2.87)	2.05(1.33-2.89)	0.679
PT(sec)	10.46 ± 0.70	10.34 ± 0.68	10.46 ± 0.70	0.973
APTT(sec)	25.09 ± 2.12	24.92 ± 2.46	25.09 ± 2.12	0.107
PTINR	0.90 ± 0.06	0.89 ± 0.06	0.90 ± 0.06	0.950
HbAlc(%)	9.26 ± 1.99	9.56 ± 2.30	9.30 ± 1.99	0.048
TC(mmol/L)	4.57 ± 1.05	4.67 ± 1.30	4.57 ± 1.05	0.110
TBIL(mmol/L)	12.20 ± 4.13	12.61 ± 5.69	12.20 ± 4.13	0.132
DBIL(mmol/L)	2.14 ± 0.81	2.21 ± 1.01	2.14 ± 0.81	0.093
AST(u/L)	18.35(14.53-22.73)	18.20(15.15-22.70)	18.35(14.53-22.73)	0.720
ALT(u/L)	19.55(12.50-27.18)	18.10(13.40-24.25)	19.55(12.50-27.18)	0.518
Scr(umol/L)	55.76 ± 18.14	57.16 ± 22.44	55.76 ± 18.14	0.682
BUN(mmol/L)	6.05 ± 1.65	6.27 ± 2.32	6.06 ± 1.65	0.200
ALB(g/L)	39.92 ± 3.68	39.58 ± 4.38	39.92 ± 3.68	0.155
FPG(mmol/L)	8.15 ± 3.00	8.42 ± 3.36	8.15 ± 3.00	0.351
ALP(u/L)	85.23 ± 28.20	82.18 ± 23.75	85.23 ± 28.20	0.309
HDL-C(mmol/L)	1.15 ± 0.30	1.16 ± 0.34	1.15 ± 0.30	0.540
LDL-C(mmol/L)	2.97 ± 0.76	3.06 ± 0.94	2.97 ± 0.76	0.185
CHE(KU/L)	8.33 ± 1.55	8.10 ± 1.98	8.33 ± 1.55	0.027
UA(umol/L)	284.22 ± 80.11	272.46 ± 73.70	284.22 ± 80.11	0.219
TG(mmol/L)	1.40(0.98-2.11)	1.35(0.97-2.03)	1.40(0.98-2.11)	0.605
DPVD, n (%)				0.727
No	124(31.2)	88(31.8)	369(30)	
Yes	273(68.8)	189(68.2)	84(70)	
DKD, n (%)				0.852
No	322(81.1)	224(80.9)	98(81.7)	
Yes	75(18.9)	53(19.1)	22(18.3)	

BMI, body mass index; WBC, white blood cell; MONO, monocyte; LYM, lymphocyte; NEUT, neutrophile granulocyte; RBC, red blood cell; Hb, hemoglobin; PLT, platelet; FT3, free triiodothyronine; FT4, free thyroxine; TSH, thyroid stimulating hormone; PT, prothrombin time; APTT, activated partial thromboplastin time; PTINR, prothrombin time international normalized ratio; HbAlc, glycated hemoglobin A1c; TC, total cholesterol; TBIL, total bilirubin; DBIL, direct bilirubin; AST, aspartate aminotransferase; ALT, alanine aminotransferase; Scr, serum creatinine; BUN, blood urea nitrogen; ALB, albumin; FPG, fasting plasma glucose; ALP, alkaline phosphatase; HDL-C, high-density lipoprotein cholesterol; LDL-C, low-density lipoprotein cholesterol; CHE, cholinesterase; UA, uric acid; TG, triglyceride; DPVD, diabetic peripheral vascular disease; DKD, diabetic kidney disease.

### Characteristics’ selection

In this research, both univariate and multivariate logistic regression analyses were applied to assess the clinical variables in patients having T2DM and DPN, as detailed in **Table 2**. The forward-backward method of logistic regression analysis indicated that factors such as duration of disease, levels of TBIL, TG, and the presence of DPVD independently influenced the occurrence of DPN in individuals with T2DM, demonstrating significant associations (P < 0.05).

**Table 2:** Univariate and multivariate logistic regression analyses for patients with T2DM.

Variables	OR(95%CI)	P value	OR(95%CI)	P value
sex,n,%	1.21(0.74-2.00)	0.449		
age(years)	1.05(1.03-1.07)	<0.001	1.02(0.99-1.04)	0.157
duration(years)	1.14(1.09-1.19)	<0.001	1.10(1.05-1.15)	<0.001
BMI(kg/^2^)	0.96(0.91-1.02)	0.225		
WBC(×10^9/L)	0.88(0.76-1.01)	0.068		
MONO(×10^9/L)	0.61(0.12-3.01)	0.543		
LYM(×10^9/L)	0.61(0.41-0.92)	0.017		
NEUT(×10^9/L)	0.90(0.76-1.07)	0.247		
RBC(×10^12/L)	0.62(0.41-0.95)	0.029		
Hb(g/L)	0.99(0.98-1.00)	0.123		
PLT(×10^9/L)	0.99(0.98-1.00)	0.018		
25(OH)D3(ng/mL)	1.01(0.99-1.02)	0.358		
FT3(pmol/L)	0.76(0.58-1.00)	0.052		
FT4(pmol/L)	0.95(0.87-1.03)	0.203		
TSH(mIU/L)	1.03(0.96-1.10)	0.385		
PT(sec)	1.00(0.70-1.43)	0.988		
APTT(sec)	0.98(0.89-1.08)	0.699		
PTINR	1.81(0.04-88.22)	0.765		
HbAlc(%)	1.05(0.95-1.17)	0.33		
TC(mmol/L)	0.90(0.74-1.08)	0.246		
TBIL(mmol/L)	0.95(0.91-1.00)	0.034	0.95(0.90-1.00)	0.045
DBIL(mmol/L)	0.86(0.68-1.09)	0.217		
AST(u/L)	0.96(0.94-0.99)	0.006		
ALT(u/L)	0.98(0.96-0.99)	0.005		
Scr(umol/L)	1.00(0.99-1.01)	0.413		
BUN(mmol/L)	1.10(0.98-1.24)	0.108		
ALB(g/L)	0.98(0.93-1.04)	0.516		
FPG(mmol/L)	1.00(0.93-1.07)	0.901		
ALP(u/L)	1.00(0.99-1.01)	0.564		
HDL-C(mmol/L)	1.22(0.57-2.59)	0.608		
LDL-C(mmol/L)	0.93(0.72-1.20)	0.573		
CHE(KU/L)	0.85(0.75-0.96)	0.012		
UA(umol/L)	1.00(0.98-1.00)	0.109		
TG(mmol/L)	0.74(0.61-0.89)	0.002	0.74(0.6-0.91)	0.004
DPVD, n (%)				
No				
Yes	2.85(1.69-4.81)	<0.001	1.99(1.03-3.86)	0.042
DKD, n (%)				
No				
Yes	2.11(1.07-4.16)	0.032		

BMI, body mass index; WBC, white blood cell; MONO, monocyte; LYM, lymphocyte; NEUT, neutrophile granulocyte; RBC, red blood cell; Hb, hemoglobin; PLT, platelet; FT3, free triiodothyronine; FT4, free thyroxine; TSH, thyroid stimulating hormone; PT, prothrombin time; APTT, activated partial thromboplastin time; PTINR, prothrombin time international normalized ratio; HbAlc, glycated hemoglobin A1c; TC, total cholesterol; TBIL, total bilirubin; DBIL, direct bilirubin; AST, aspartate aminotransferase; ALT, alanine aminotransferase; Scr, serum creatinine; BUN, blood urea nitrogen; ALB, albumin; FPG, fasting plasma glucose; ALP, alkaline phosphatase; HDL-C, high-density lipoprotein cholesterol; LDL-C, low-density lipoprotein cholesterol; CHE, cholinesterase; UA, uric acid; TG, triglyceride; DPVD, diabetic peripheral vascular disease; DKD, diabetic kidney disease.

### Development of an individualized prediction model

**Figure 2:**
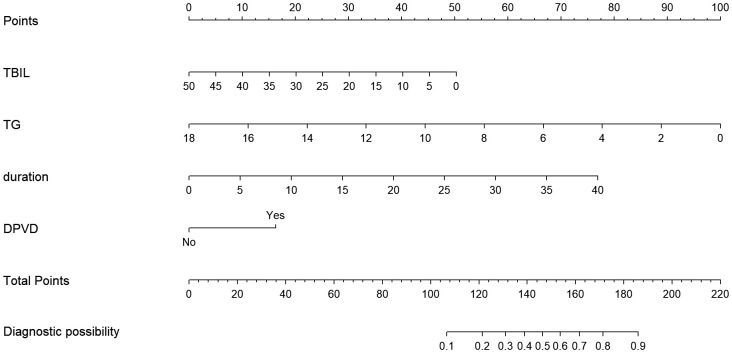
The nomogram model for quantifying individual risk of DPN in patients with T2DM.

The study utilized univariate and multivariate logistic regression to pinpoint four key independent predictors. Among these, TBIL and TG acted as protective elements against DPN, whereas the remaining two were identified as risk contributors (refer to **Table 2**). This research led to the creation of a model encompassing these predictors, depicted in the form of a nomogram (refer to **Figure 2**). Each predictor’s score was assigned according to a specific scale within the nomogram, linked to its respective risk factor. Summing these scores yielded an overall score, which was then used to estimate the likelihood of DPN occurrence. This overall score ranged from 0 to 220, with the associated risk level varying between 0.1 and 0.9. Essentially, an elevated total score indicated an increased risk of DPN among T2DM patients.

### Validation of the nomogram

**Figure 3:**
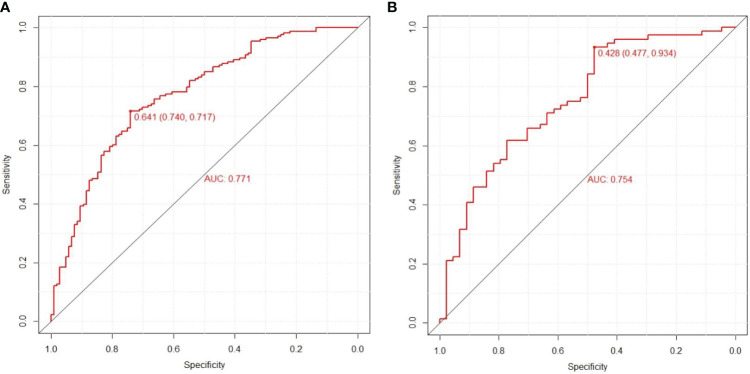
Prediction performance of the model. Receiver operating characteristic (ROC) curve plot in the training cohort **(A)**; ROC curve plot in the validation cohort **(B)**; AUC, the area under the ROC.

**Figure 4:**
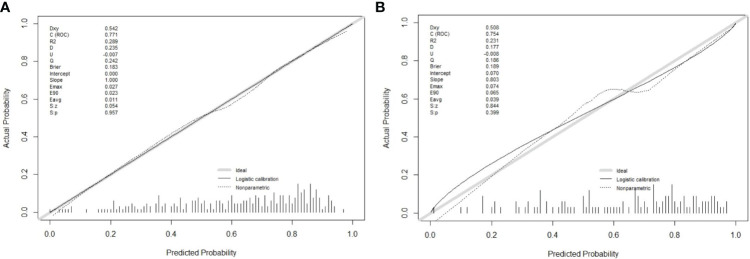
Calibration curve plot in each cohort. **(A)** the training cohort; **(B)** the validation cohort.

**Figure 5:**
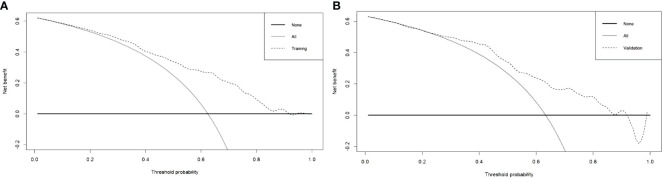
Decision curve analysis of training cohort **(A)** and validation cohort **(B)** for the risk of DPN in patients with T2DM.

In this research, the nomogram exhibited significant predictive accuracy, with AUC values of 0.771 (95% CI: 0.714-0.828) for the training group (as shown in **Figure 3A**) and 0.754 (95% CI: 0.663-0.845) for the validation group (as indicated in **Figure 3B**). The model’s accuracy is visually represented in the calibration plots by a black line, where its closeness to the diagonal gray line reflects higher predictive precision. Both the training (illustrated in **Figure 4A**) and validation (shown in **Figure 4B**) cohort calibration plots demonstrated a strong correlation between the model’s estimated probabilities and the actual data. The calibration analysis, assessed via HL tests, indicated Chi-square values of 7.689 for the training cohort and 6.612 for the validation cohort, with P-values of 0.609 and 0.565, respectively, suggesting a reliable fit of the model. Additionally, DCA emphasized the nomogram’s clinical utility in estimating DPN risk across various probability thresholds, as evidenced in the training (**Figure 5A**) and validation (**Figure 5B**) cohorts.

## Discussion

Considering the detrimental impact of DPN, it is crucial for healthcare professionals to focus on its management, aiming to reduce its prevalence among T2DM patients. Recent studies have indicated that nomograms are effective in intuitively predicting the risk of diseases. This research was centered on creating and validating a new nomogram, tailored for estimating the likelihood of DPN in individuals with T2DM. This nomogram is a significant asset in predicting and identifying individual risk factors related to DPN. In this study, logistic regression and forward-backward stepwise regression methods were utilized to examine the clinical data of T2DM patients with concurrent DPN. This analysis highlighted TBIL, TG, diabetes duration, and DPVD as crucial independent predictors for DPN. This study’s nomogram model included these four critical predictors. The model’s effectiveness was evidenced by generating ROC curves, with the training and validation cohorts showing AUCs of 0.771 (95% CI, 0.714, 0.828) and 0.754 (95% CI, 0.663, 0.845), respectively, indicating the model’s robust capacity for discrimination. The calibration curve further confirmed the model’s precision in estimating the risk of DPN. At the sama time, the area under the curve (AUC) of the column chart model designed by Wanli Zhang et al. was 0.763 in the training queue and 0.755 in the validation queue (11). Jing Yang et al. found serum creatinine (Scr), hypertension, glycosylated hemoglobin A1c (HbA1c), blood urea nitrogen (BUN), body mass index (BMI), triglycerides (TG), and diabetic peripheral neuropathy (DPN) Can serve as a key factor for this prediction model. The Harrell’s C-indexes were 0.773 (95% CI: 0.726-0.821) and 0.758 (95% CI: 0.679-0.837) in the training and validation sets, respectively (12). The accuracy of the prediction model in this study is almost comparable to that of existing prediction models, but the key factors of the prediction model in this study are only 4, which is less than the key factors of existing models. Therefore, overall, the predictive model of this study is superior to existing predictive models. Additionally, the DCA decision curves supported the model’s efficacy in prediction, underscoring its reliability as a tool for assessing DPN risk.

Numerous studies have highlighted that the length of time a patient has had T2DM, combined with DPN (13–16), is a crucial determinant. As the duration of diabetes increases, the likelihood of developing DPN also rises. Notably, individuals who have had T2DM for five years or more tend to experience gradual declines in their vibration perception threshold, reaction time, and balance (13). A particular cross-sectional study underscored a notable positive correlation between the length of diabetes and the prevalence of DPN, suggesting that DPN progression is closely tied to how long one has had diabetes, typically spanning 8 to 16 years (14). Amelia R et al. also observed that individuals diagnosed with DM for more than five years have a higher propensity for DPN (15). The prolonged suffering from DM escalates the risk and exacerbates nerve cell damage over time (15, 16). This finding helps clarify why the duration of diabetes was selected as a key variable in our study’s final model.

In the past few years, there has been a growing focus on the role of TG levels in predicting DPN. Research increasingly shows that elevated triglycerides, or hypertriglyceridemia, heighten the risk of developing DPN by nearly four times, independently of blood glucose levels. This risk is often linked to the degeneration of small unmyelinated nerve fibers (17–20). Increasing levels of TG are considered a primary factor in the advancement of DPN, attributed to heightened levels of triglyceride metabolites (plasma free fatty acids). These acids instigate inflammatory reactions and oxidative stress within sensory neurons, potentially resulting in substantial cellular harm. Such damage encompasses endoplasmic reticulum stress, mitochondrial impairment, and permanent neuronal injury (19). Consequently, TG is a crucial factor in our predictive model. Additionally, this study has noted that increased TBIL levels might play a protective role in DPN’s development (21–24). The exact biological mechanisms connecting TBIL levels to DPN remain speculative. However, it is hypothesized that bilirubin’s strong antioxidant properties might offer direct nerve tissue protection by mitigating oxidative stress (21–24). Furthermore, bilirubin’s anti-inflammatory effects could contribute to reducing nerve damage (22, 23), and its neuroprotective impact might be linked to changes in enzyme activities within the bilirubin metabolism pathway (22, 23). Thus, incorporating TBIL levels into the model is essential for accurately predicting DPN.

Within our final model, three factors – duration, TG, and TBIL – are already established as being correlated with DPN based on prior research. Conversely, DPVD’s association with DPN is a novel discovery. This study is the inaugural one to identify DPVD as a predictive factor for DPN, possibly linked to several indirect influences, particularly oxidative stress and lipid metabolism (25, 26). Therefore, DPVD may be significantly associated with the risk of DPN in T2DM patients.

In conclusion, this research introduces a clear and concise risk assessment tool based on four clinical parameters, proving its effectiveness in forecasting the risk of DPN in T2DM patients. This model can be seamlessly integrated into clinical practice by healthcare professionals for assessing DPN risk in patients with T2DM. Furthermore, it offers a significant foundation for the creation of upcoming clinical trials that focus on thwarting the onset of DPN in individuals suffering from type 2 diabetes.

Nevertheless, there were certain constraints in this study. Primarily, it was a retrospective study based in a single center. For improved clinical relevance and to achieve broader external validation, it is advisable for subsequent research to adopt a prospective approach, incorporating data from various centers. Additionally, the sample size in this study was relatively small, and we relied solely on internal validation to assess the model’s accuracy and effectiveness. Future research should encompass larger sample sizes and incorporate a broader range of variables to corroborate our findings. Another limitation is that, for the sake of clinical practicality, the model only incorporated commonly used laboratory and clinical assessment indicators, excluding newer biomarkers such as serum periostin.

## Conclusions

This research methodically developed and validated an innovative nomogram designed to predict DPN risk in T2DM patients. This easy-to-use scoring model equips medical practitioners with an efficient means to optimize the management of T2DM patients, providing a practical solution for quick and tailored clinical decision-making.

## Data availability statement

The original contributions presented in the study are included in the article/supplementary material, frther inquiries can be directed to the corresponding author/s.

## Ethics statement

The studies involving humans were approved by the ethics committee of the Suqian First Hospital. The studies were conducted in accordance with the local legislation and institutional requirements. The participants provided their written informed consent to participate in this study.

## Author contributions

LL: Writing – original draft, Validation, Software, Methodology, Investigation. XL: Writing – original draft, Validation, Formal analysis. CC: Writing – original draft, Visualization, Data curation. QX: Writing – original draft, Methodology, Data curation. JL: Writing – review & editing, Writing – original draft, Visualization, Funding acquisition, Formal analysis, Data curation.
